# ﻿Taxonomic study of the genus *Campylomyza* Meigen (Diptera, Cecidomyiidae) in Korea with descriptions of seven new species

**DOI:** 10.3897/zookeys.1223.128062

**Published:** 2025-01-09

**Authors:** Daseul Ham, Yeon Jae Bae

**Affiliations:** 1 Department of Environmental Science and Ecological Engineering, Graduate School, Korea University, Seoul, Republic of Korea Korea University Seoul Republic of Korea; 2 Species Diversity Research Division, Biodiversity Research Department, National Institute of Biological Resources, Incheon, Republic of Korea National Institute of Biological Resources Incheon Republic of Korea

**Keywords:** Distributional new records, Korea, Micromyinae, Mycophagous cecidomyiids, new species

## Abstract

The genus *Campylomyza* Meigen, 1818, belongs to the subfamily Micromyinae (Diptera, Cecidomyiidae). The genus, comprising 40 species, is best known in the Palearctic Region. To date, four species are recorded in Korea: *Campylomyzaappendiculata*, *C.flavipes*, *C.furva*, and *C.spinata*. Based on our field investigations from 2017 to 2020, five species are newly recorded from Korea (*C.abjecta*, *C.aborigena*, *C.cornuta*, *C.cavitata*, and *C.cingulata*) and seven new species are described (*C.ambulata***sp. nov.**, *C.angusta***sp. nov.**, *C.convexa***sp. nov.**, *C.cornigera***sp. nov.**, *C.hori***sp. nov.**, *C.odae***sp. nov.**, and *C.salicia***sp. nov.**), based on morphological identification and molecular analyses. Detailed morphological and molecular data, including mitochondrial *COI* sequences are provided, with species diagnosis, descriptions, and keys for identification of those species.

## ﻿Introduction

Cecidomyiidae, a family within the order Diptera, is known for its diverse ecological roles and is considered part of the “Dark Taxa” with other Dipteran families such as Chironomidae, Phoridae, and Sciaridae, owing to their high species diversity (Chimeno et al. 2022). The term “Dark Taxa” refers to groups with a high number of undescribed species, reflecting their status as significantly understudied, which in turn limits our understanding of their taxonomy. Ecological traits of Cecidomyiidae vary among species, and can be categorized into three groups based on their larval feeding behavior: fungivorous, phytophagous, and predatory (Yukawa 2005). Additionally, Cecidomyiidae can be further classified into six subfamilies based on morphological traits: Catotrichinae, Lestremiinae, Micromyinae, Winnertziinae, Porricondylinae, and Cecidomyiinae (Gagné and Jaschhof 2021). The genus *Campylomyza*, belonging to the fungivorous group within the Micromyinae subfamily, typically inhabits decaying plant roots, leaves, and fungal fruiting bodies in soil (Mamaev and Krivosheina 1993). These small flies, measuring between 1.0 and 1.8 mm, are known for their swarming behavior, mainly observed for mating purposes during the cooler months (early April, May, and October), particularly when temperatures range from 16 °C to 24 °C (Kanmiya 1985, 1996, 1999; Kanmiya and Yukawa 2020).

Taxonomic studies of *Campylomyza* began in 1818 with Meigen’s description of four species (*C.aceris* Meigen, 1818, *C.atra* Meigen, 1818, *C.bicolor* Meigen, 1818, and *C.flavipes* Meigen, 1818) (Meigen 1818). Westwood (1840) provided a concise description of *Campylomyza* with a catalog-like list of insects, designating *C.flavipes* as the type species. However, the original descriptions were insufficient for accurate identification. Subsequently, Edwards and Jaschhof provided revised characters and detailed descriptions of *Campylomyza* species (Edwards 1938a, b; Jaschhof 1998a; Jaschhof and Jaschhof 2009). Mamaev (1963, 1998), Jaschhof (1998a, 2015), and Jaschhof and Jaschhof (2009) made significant contributions to the taxonomy, describing the majority of known species. *Campylomyza* species exhibit remarkably high morphological similarities and display complex diversity with potentially numerous cryptic species (Jaschhof 2015). Jaschhof and Jaschhof (2009) subdivided *Campylomyza* into seven groups (*alpina*, *bicolor*, *cornuta*, *flavipes*, *ormerodi*, *serrata*, and incertae sedis group) based on male genitalia morphology. This reclassification revealed distinct species within putatively highly variable species, such as *C.flavipes*, *C.ormerodi* (Kieffer, 1913), and *C.serrata* Jaschhof, 1998. For instance, the *C.flavipes* complex was split into five species, *C.ormerodi* into six species, and *C.serrata* into five species (Jaschhof and Jaschhof 2009; Jaschhof 2015).

Cecidomyiidae is recognized as one of the most species-rich taxa, with an estimated 1.8 million species worldwide, yet only 6,651 species have been recorded to date (Gagné and Jaschhof 2021; Hebert et al. 2016). In South Korea, only 117 species have been documented, including 68 mycophagous cecidomyiids (Han 2021). The genus *Campylomyza* has been primarily studied within the Palearctic region, with limited research conducted in other biogeographic regions, including the Nearctic. To date, 40 *Campylomyza* species are recognized globally, comprising 39 species from the Palearctic region, two from the Nearctic region, and one from the Oriental region. Two species occur in both the Nearctic and Palearctic regions: *C.dilatata* Felt, 1907 and *C.flavipes*. Among them, five species are known from the Russian Far East, four species from Japan, and one species from China (Mamaev 1998; Gagné and Jaschhof 2021; Jaschhof 1998a, 2015; Jaschhof and Jaschhof 2009). In Korea, only four *Campylomyza* species have been previously recorded (Ham et al. 2019, 2020). In this study, we report five species newly recorded in South Korea and describe seven species new to science. Consequently, the global total of described *Campylomyza* species has increased to 47, with South Korea accounting for 16 of these, as determined through morphological analysis and mitochondrial Cytochrome Oxidase subunit I (mtCOI) sequencing. Detailed descriptions, diagnoses, and illustrations are provided for both the new and redescribed species. Our study aims to enhance our knowledge of the *Campylomyza* fauna in South Korea and contribute to various scientific fields, including forest ecology and the study of invasive species (Kang et al. 2023). Given the ongoing rapid climate change, it is crucial to thoroughly study organisms in their existing habitats to predict and understand potential environmental changes.

## ﻿Materials and methods

### ﻿Taxon sampling and morphological identification

**Figure 1.**
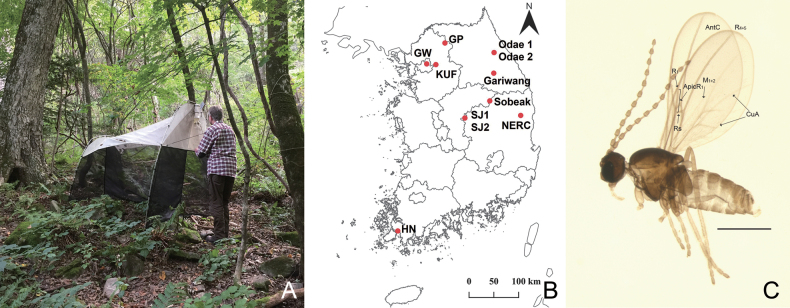
**A** Malaise trap in Odae mountain (17 Sept. 2019) **B** map of sampling sites for specimens collected in the present study. For abbreviations of locations, see Table 1. The map was prepared using QGIS 3.28.1 (https://www.qgis.org/ko/site/) **C** male adult habitus of *Campylomyzaaborigena* excluding genitalia and two legs. Scale bar = 0.5 mm. Abbreviations: AntC: anterior cubitus, R_1_: anterior branch of radius, ApicR_1_: apical part of R_1_, R_4+5_: third branch of radius, M_1+2_: combined form of the first and second branches of the media, CuA: anterior branch of cubitus.

All samples were collected between 2017 and 2020 using Malaise traps (Fig. 1A) located in Gyeonggi-do, Gangwon-do, Gyeongsangbuk-do, and Jeollanam-do, Korea. The locations where Malaise traps were installed are indicated by abbreviations (Fig. 1B, Table 1, Suppl. material 1: table S1), and the corresponding habitat information is provided as follows:

**Table 1.** Location data of collected Korean *Campylomyza* species and species occurrence by region.

**Abbreviation**	**Location**	**Species**
**Gariwang**	Gangwon-do, Jeongseon-gun, Jeongseon-eup, Hoedong-ri, 870, Gariwangsan Recreational Forest	*C.hori* sp. nov.
**GP**	Gyeonggi-do, Gapyeong-gun, Buk-myeon, Garimgyo (bridge name)	*C.ambulata* sp. nov.
**GW**	Seoul, Seongbuk-gu, Bugaksan-ro, Mt. Gaewun	*C.ambulata* sp. nov.
**HN**	Jeollanam-do, Haenam-gun, Hwangsan-myeon, Oeip-gil	*C.cornuta*
**KUF**	Gyeonggi-do, Namyangju-si, Wabu-eup, Dosim-gil, Korea University’s farm to practice	*C.abjecta*
		*C.appendiculata*
		*C.cornigera* sp. nov.
		*C.convexa* sp. nov.
		*C.furva*
**NERC**	Gyeongsangbuk-do, Yeongyang-gun, Yeongyang-eup, Gowol-gil, 23, National Endangered Species Restoration Center	*C.abjecta*
		*C.ambulata* sp. nov.
		*C.cornigera* sp. nov.
		*C.flavipes*
		*C.furva*
		*C.furva*
		*C.salicia* sp. nov.
		*C.spinata*
**Odae 1**	Gangwon-do, Pyeongchang-gun, Jinbu-myeon, Odaesan-ro, Mt. Odae, small valley before So-Myeong valley	*C.angusta* sp. nov.
		*C.cavitata*
		*C.cornigera*
		*C.cingulata*
**Odae 2**	Gangwon-do, Pyeongchang-gun, Jinbu-myeon, Odaesan-ro, Mt. Odae, the road before temple (Buk-Dae-Mi-Reuk-Am)	*C.aborigena*
		*C.ambulata*
		*C.flavipes*
		*C.flavipes*
		*C.odae* sp. nov.
**SJ 1**	Gyeongsangbuk-do, Sangju-si, Hwabuk-myeon, Ipseok-ri	*C.abjecta*
		*C.appendiculata*
		*C.spinata*
**SJ 2**	Gyeongsangbuk-do, Sangju-si, Hwabuk-myeon, Ipseok-ri	*C.abjecta*
		*C.ambulata* sp. nov.
		*C.appendiculata*
**Sobaek**	Gyeongsangbuk-do, Yeongju-si, Punggi-eup, Sucheol-ri, Mt. Sobaek	*C.aborigena*
		*C.hori* sp. nov.

**Gariwang** A deciduous forest composed of medium-aged trees of the 6^th^ class. The Malaise trap was placed beside a hiking trail on slightly sloped terrain. The forest floor consisted of broadleaf litter mixed with stones larger than 30 cm in diameter. The surrounding area was highly humid, with abundant organic matter and a high presence of spiders and flies observed between the stones.

**GP** The Malaise trap was installed along a valley near oak and Korean pine trees, classified as 5^th^ class or higher in maturity. This location was situated close to residential areas.

**GW** An urban-managed forest. The Malaise trap was placed near a trail that occasionally becomes a stream during heavy rainfall. The forest is mainly composed of medium-sized deciduous trees of the 5^th^ class or higher.

**HN** Located in the southern part of the Korean Peninsula near the coastline. The Malaise trap was installed close to farmland in a coniferous forest composed of small trees (2^nd^ class black pine).

**KUF** The Malaise trap was placed in the experimental farm of Korea University, surrounded by pine trees.

**NERC** The Malaise trap was installed at the National Ecology Research Center in Yeongyang, next to a large river and reed field.

**Odae1** Situated in Mt. Odae National Park, a protected area. The Malaise trap was placed next to a valley in a deciduous forest with medium-aged trees classified as 5^th^ class or higher.

**Odae2** Located at an altitude of more than 1,200 meters, the site features a deciduous forest with medium-sized trees (5^th^ class). Due to the high elevation, the trees were relatively shorter, and the area was comparatively less humid.

**SJ1, SJ2** The Malaise traps were placed next to farmland near Mt. Sokri National Park.

**Sobaek** A protected area within Mt. Sobaek National Park. The forest consists of medium-sized deciduous trees, classified as 4^th^ class or higher.

Specimens were dissected, with one or two legs removed, and preserved in 100% alcohol for molecular analysis. The samples were cleared in Creosote reagent and subsequently mounted in Canada balsam under a stereomicroscope (Olympus SZ51, Tokyo, Japan) following the methods described in relevant literature (Jaschhof and Jaschhof 2009, 2013). The specimens were examined using a bright-field and optical microscopy (Olympus BX50, Tokyo, Japan). Microscopic images of the specimens were captured using a Nikon D750 camera (Tokyo, Japan) attached to an optical microscopy (Olympus BX50). The images were taken at different focal planes and stacked using Helicon Focus software® (Helicon Soft, Ltd). Drawings were created using a drawing tube (Olympus U-DA). The terminology used in this study generally follows Jaschhof and Jaschhof (2009) and Jaschhof and Fitzgerald (2016). We list species in alphabetical order. The illustrations and photographs include arrows to indicate the diagnostic features. The body length was measured as the horizontal length of the head, thorax, and abdomen, excluding the antenna.

Voucher material is deposited in the collections of the Korean Entomological Institute, Korea University (**KU**), and the National Institute of Biological Resources, Incheon, Korea (**NIBR**).

In a previous abstract presented at ‘2024 Spring conference of KSAE & ESK (Korean Society of Applied Entomology & The Entomological Society of Korea)’, the species names (*C.angusta* sp. nov., *C.ambulata* sp. nov., *C.convexa* sp. nov., *C.cornigera* sp. nov., *C.hori* sp. nov., and *C.odae* sp. nov.) were mentioned but not formally described (Ham et al. 2024; https://db.koreascholar.com/Article/Detail/433436). According to the International Code of Zoological Nomenclature (ICZN), those names were considered nomina nuda, as no formal description was provided at that time. This manuscript provides the first formal descriptions of these species, fulfilling the requirements of valid publication and nomenclature.

### ﻿DNA extraction, sequencing, and alignment

Total genomic DNA was extracted from typically two or three legs of adult male specimens of *Campylomyza* using the DNeasy Blood and Tissue Kit (Qiagen, Hilden, Germany) according to the manufacturer’s instructions. For PCR amplification of the mitochondrial cytochrome oxidase subunit I (mtCOI, ~ 676 bp), we utilized the primer set: Forward: LCO1490 (5’-GGT CAA CAA ATC ATA AAG ATA TTG G-3’; Folmer et al. 1994) and Reverse: COIA (5′-CCC GGT AAA ATT AAA ATA TAA ACT TC-3′; Funk et al. 1995). Amplification was performed using AccuPower PCR Premix (Bioneer, Daejeon, Republic of Korea) following standard protocols. The PCR conditions consisted of an initial denaturation at 95 °C for 3 min, followed by 35 cycles of denaturation at 95 °C for 30 s, annealing at 50 °C for 30 s, extension at 72 °C for 1 min, and a final extension at 72 °C for 10 min. Successfully amplified PCR products were checked on 1.2% Agarose gels and were purified and sequenced at BIONICS, Inc. (Seongdong-gu, Seoul, Republic of Korea). The sequences obtained were compared using Geneious Prime v. 2023.1.1 and deposited in the NIBR (National Institute of Biological Resources; https://species.nibr.go.kr/index.do) and GenBank (https://www.ncbi.nlm.nih.gov/) with accession numbers.

### ﻿DNA barcode sequence analysis and delimitation

A total of 33 *COI* sequences were analyzed as DNA barcodes in this study (Suppl. material 1: table S2). These include 31 sequences from 15 *Campylomyza* species found in Korea, one sequence of *C.flavipes* from GenBank, and one sequence from the outgroup species, *Peromyiatrifida* Jaschhof, 2001. For phylogenetic analyses, we constructed the neighbor-joining (NJ) under the Kimura-2-parameter (K2P) model (Kimura 1980) with 1,000 bootstrap replicates using MEGA X (Kumar et al. 2018). Pairwise comparison of uncorrected genetic distances (*p*-distances) was used to estimate sequence divergence, and the complete deletion option was applied in MEGA X. Also, we used Automatic Barcode Gap Discovery (ABGD) as species delimitation method for estimating the number of Molecular Operational Taxonomic Units (MOTUs) (Puillandre et al. 2012). The ABGD assessment was conducted online using the provided website (https://bioinfo.mnhn.fr/abi/public/abgd/), utilizing the Jukes-Cantor (JC69), Kimura 2-parameter (K2P), and uncorrected distance (p-distance) models, with a relative gap width set at X = 1.5 (Oh et al. 2022).

## ﻿Taxonomic accounts

**Family Cecidomyiidae**

**Subfamily Micromyinae**

### ﻿Genus Campylomyza

Taxon classificationAnimaliaDipteraCecidomyiidae

Meigen, 1818

AC7C741F-6FBB-5CE8-84AF-FC6BEB07096E

Fig. 1C

Campylomyza Meigen, 1818: 101; Westwood 1840: 126; Jaschhof 1998b: 136; Jaschhof and Jaschhof 2009: 89.

#### Type species.

*Campylomyzaflavipes* Meigen, 1818 (original designation by Westwood 1840). Type locality Germany.

#### Diagnosis.

The adult males of the South Korean genus *Campylomyza* can be distinguished from other mycophagous cecidomyiid taxa based on the following combination of characters [adapted from Jaschhof and Jaschhof 2009]: 1) Antenna with 12 flagellomeres; 2) Node of fourth antennal flagellomere featuring one complete and two incomplete crenulate whorls with sensory hairs with two incompletely collar-shaped sensilla distally; 3) Apical part of the R_1_ vein, located near wing tip (ApicR_1_) elongated, approximately 4–6 times the length of Rs; 4) Gonostyli strongly convex posteriorly without apical spines; 5) Aedeagal apodeme equipped with typical head-like structure; 6) Tegmen with transverse brace (i.e., H-shaped), various processes on apex.

### ﻿ Campylomyza abjecta

Taxon classificationAnimaliaDipteraCecidomyiidae

Mamaev, 1998

391D8874-F148-5D67-B6E6-E652389517BB

Campylomyza abjecta Mamaev 1998: 6.
Campylomyza abjecta Jaschhof and Jaschhof 2017: 5, fig. 2A–C (redescription).

#### Distribution.

Russia (Primorsky), Sweden, new record for South Korea.

#### Specimens examined.

Korea • 3♂♂ (slides no. 19AY-3, 7, 19AYa-9, 10); **NERC**; 10–17 Apr. 2019; Y. J. Choi, H. G. Kim leg.; deposited in KU • 1♂ (slide no. NIBRIN0000857557); **KUF**; 2–8 Apr. 2017; D. Ham leg.; deposited in NIBR • 1♂ (slide no. NIBRIN0000992636); **SJ 1**; 13 Apr. – 4 May 2019; W. G. Kim leg.; deposited in NIBR • 1♂ (slide no. NIBRIN0000992634); **SJ 2**; 8–24 Apr. 2020; W. G. Kim leg.; deposited in NIBR.

**Figure 2.**
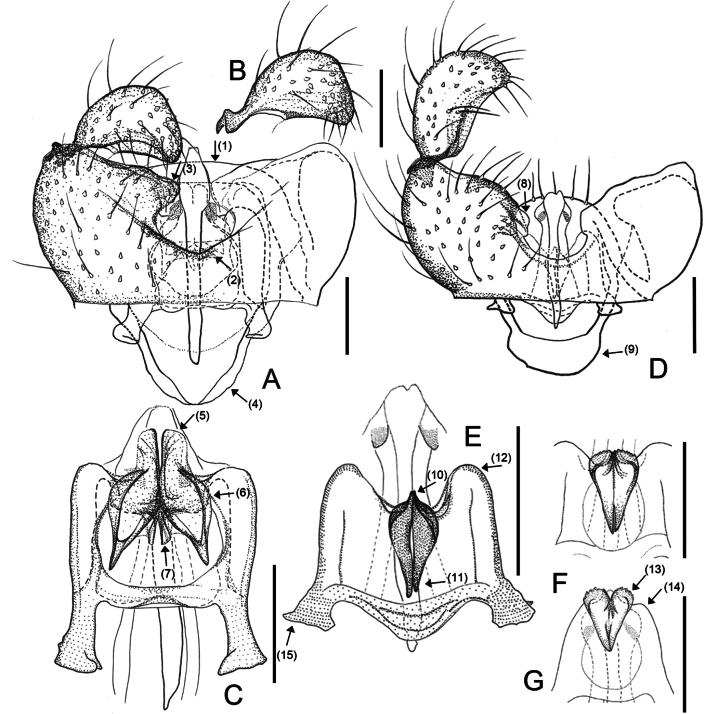
Male morphology of *Campylomyzaaborigena* Mamaev, 1998 (**A–C**) and *Campylomyzaambulata* sp. nov. (**D–G**) **A** gonocoxites, ventral view, slide no. NIBRIN0000992639 **B** gonostylus, dorsal view, slide no. NIBRIN0000992639 **C** tegmen, dorsal view, slide no. 19–38 **D** gonocoxites, ventral veiw, holotype **E–G** tegmen, dorsal view **E** holotype **F** paratype (slide no. NIBRIN0000919403) **G** paratype (slide no. 19AY-9). Scale bars: 0.05 mm.

### ﻿ Campylomyza aborigena

Taxon classificationAnimaliaDipteraCecidomyiidae

Mamaev, 1998

A46C5981-898F-53AF-A9BA-5002BB19AC85

Fig. 1C , 2A–C

Campylomyza aborigena Mamaev, 1998: 6.

#### Specimens examined.

Korea • 1♂ (slide no. NIBRIN0000992639); **Sobaek**; 6 May – 6 Jun. 2019; D. Ham, S. Park leg.; deposited in NIBR • 2♂♂ (slides no. NIBRIN0000992638, 19-38); **Odae 2**; 23 Apr. – 11 May 2019; D. Ham, S. Park leg.; deposited in NIBR.

#### Diagnosis.

*Campylomyzaaborigena* closely resembles *C.aemula* Mamaev, 1998 (inferred from the illustration in Jaschhof and Jaschhof 2009) and shares the following characteristics: 1) Tegmen with lamellate (Fig. 2C, ↓_5_), tapering apical points that are rounded and strongly sclerotized anteriorly, and weakly sclerotized posteriorly; 2) Large foliate dorsal processes (Fig. 2C, ↓_6_) with narrower, sharp points; 3) Gonostyli tapering apically and curved anteroventrally with convex apex margins (Fig. 2B). However, *C.aborigena* can be distinguished from *C.aemula* by the following characteristics: tegmen with parallel-sided apical points (Fig. 2C, ↓_5_); dorsal processes large, broad basally, pointed apically with strongly sclerotized margin (Fig. 2C, ↓_6_).

#### Measurements.

Male adult (Slide no. NIBRIN0000992639): Body length 1.454 mm. Wing length 1.484 mm. Hind leg coxa 0.170 mm; femur 0.547 mm; tibia 0.551 mm; tarsomere I 0.307 mm; tarsomere II 0.164 mm; tarsomere III 0.130 mm; tarsomere IV 0.067 mm; tarsomere V 0.062 mm.

#### Redescription.

Male adult. ***Head*.** Postocular bristles four or five. Antenna with 12 flagellomeres. Neck of fourth antennal flagellomeres as long as node. Node with one complete and two incomplete crenulate whorls with sensory hairs, two incompletely collar-shaped sensilla distally. Palpus 4-segmented; fourth segment longest. ***Thorax*.** Preepisternum with eight setae. Wing length to width ratio 2.44, AntC ending beyond R_4+5_ but before reaching M_4_; ApicR_1_ 3.23× length of Rs; CuA separated (Fig. 1C). Tarsomere I longer than tarsomere II. Claws sickle-shaped, toothed; empodia longer than claws; pubescent. ***Terminalia*.** Tg9 slightly tapered towards apex (Fig. 2A, ↓_1_). Gonocoxites emarginated broad U-shaped ventrally. (Fig. 2A, ↓_2_); ventromedial portion swollen, pronounced (Fig. 2A, ↓_3_); dorsal transverse bridge narrower to apex, extending far beyond ventrobasal margin (Fig. 2A, ↓_4_). Gonostyli curved anteroventrally; apical margin strongly convex; medial portion excavated; setae becoming denser towards apex. On tegmen, apical points long, lamellate, rounded apically (Fig. 2C, ↓_5_); dorsal processes spoon-shaped with hollow in the center and strongly sclerotized apex; directed anteriorly (Fig. 2C, ↓_6_). Mesal processes short, sclerotized (Fig. 2C, ↓_7_). Tegmen shoulders inconspicuous.

#### Distribution.

Russia (Primorsky), new to South Korea.

#### Remarks.

*Campylomyzaaborigena* Mamaev, 1998 was originally described based on a single specimen collected in Far East Russia in 1964. Mamaev’s description was limited to just seven lines of text, without any accompanying drawings or photographs. However, thanks to the observations made by Dr. Mathias Jaschhof on the holotype specimen of *C.aborigena* in the Zoological Museum of Moscow State University in 2006, we now know that the Korean species is the same as the Russian *C.aborigena*. This is significant because it provides further evidence supporting the existence of *C.aborigena*, with the Korean finding being only the second record except for the holotype. Mamaev often described species based on a single specimen without proper illustration or depiction. Therefore, the discovery of this species in Korea and the possibility of obtaining additional specimens are of great importance for further supporting Mamaev’s species concept and advancing the taxonomy of mycophagous cecidomyiids.

### ﻿ Campylomyza ambulata sp. nov.

Taxon classificationAnimaliaDipteraCecidomyiidae

69FBE51B-451C-57A8-8F21-584882A89FC7

https://zoobank.org/EFD8D1BC-5BE8-4C74-AF49-5B7B59531A0D

Fig. 2D–G

#### Type material examined.

***Holotype***: Korea • 1♂ (slide no. 19AYa-11); Gyeongsangbuk-do, Yeongyang-gun, Yeongyang-eup, Gowol-gil, 23, National Endangered Species Restoration Center **(NERC)**; 10–17 Apr. 2019; Y. J. Choi, H. G. Kim leg.; deposited in KU. ***Paratypes***: Korea • 6♂♂ (slides no. 19AY-4, 8, 9, 11, 12, 14, 19AYa-6, 12); same data and deposition as holotype • 1♂ (slide no. 19AZ-10); **NERC**; 3–10 Apr. 2019; Y. J. Choi, H. G. Kim leg.; deposited in KU • 1♂ (slide no. NIBRIN0000919403); **NERC**; same data as for preceding; deposited in NIBR.

#### Other material examined.

Korea • 2♂♂ (slides no. NIBRIN0000992627, NIBRIN0000992628); **Odae 2**; 23 Apr. – 11 May 2019; D. Ham, S. Park leg.; deposited in NIBR • 1♂ (slide no. HDS-674); **GW**; 8 Nov. 2017; D. Ham leg.; deposited in KU • 1♂ (21AE-2-2); **GP**; 28 Apr. – 5 May 2019; Y. J. Bae leg.; deposited in KU • 1♂ (21AG-1-5); **SJ 2**; 8–24 Apr. 2020; W. G. Kim leg.; deposited in KU.

#### Diagnosis.

*Campylomyzaambulata* sp. nov. can be distinguished from other species in the *flavipes* group found in Korea through the following characteristics: 1) gonostyli curved anteroventrally, excavated ventromesally with denser setae towards the apex; 2) apical point small, short subtriangular (Fig. 2E, ↓_10_); 3) dorsal processes strongly tapering anteriorly, moveable depending on the pressure (Fig. 2E, ↓_11_); 4) shoulders of tegmen conspicuous (Fig. 2E, ↓_12_); 5) parameral apodeme short.

#### Measurements.

Male adult (holotype): Body length 1.187 mm. Wing length 1.364 mm. Hind leg coxa 0.134 mm; femur 0.480 mm; tibia 0.500 mm; tarsomere I 0.290 mm; tarsomere II 0.133 mm; tarsomere III 0.112 mm; tarsomere IV 0.071 mm; tarsomere V 0.058 mm.

#### Description.

Male adult (holotype). ***Head*.** Postocular bristles 3–5. Antenna with 12 flagellomeres. Neck of fourth antennal flagellomeres as long as node. Node with one complete and two incomplete crenulate whorls with sensory hairs, two incompletely collar-shaped sensilla distally. Palpus 4-segmented; fourth segment longest. ***Thorax*.** Preepisternum with eight setae. Wing length to width ratio 2.28, AntC ending beyond R_4+5_ but before reaching M_4_; ApicR_1_ 3.46× length of Rs; CuA separated. Tarsomere I longer than tarsomere II. Claws sickle-shaped, toothed; empodia longer than claws, pubescent. ***Terminalia*.** Tg9 tapering towards apex with five fine setae apically. Ventral emargination U-shaped; ventromedial portion of gonocoxites slightly pronounced (Fig. 2D, ↓_8_). Gonostyli with moderately convex apical margins, excavated ventromedially, narrowly rounded apically. Dorsal transverse bridge broadly rounded apically, extending beyond ventrobasal margin (Fig. 2D, ↓_9_). On tegmen, apical points small, subtriangular, lamellate (Fig. 2E, ↓_10_), dorsal processes long, strongly tapering towards apex beyond midlength, blunt apically (Fig. 2E, ↓_11_). Tegmen shoulders well-developed (Fig. 2E, ↓_12_), Parameral apodeme short (Fig. 2E, ↓_15_).

#### Variation.

We observed significant variation concerning apical points and tegmen shoulders (Fig. 2F–G). Apical points bulged with round serrated surfaces (Fig. 2G, ↓_13_); Shoulders inconspicuous, when almost in the same position or lower than apical points of tegmen (Fig. 2G, ↓_14_). Dorsal processes moveable apically.

#### Etymology.

The species epithet *ambulata* is derived from the Latin word *ambulātus*, which means ambulatory, referring to the movable nature of the dorsal processes.

### ﻿ Campylomyza angusta sp. nov.

Taxon classificationAnimaliaDipteraCecidomyiidae

D7178C00-6538-5A48-BF28-E24F28153231

https://zoobank.org/69317C2B-444C-466F-98FE-F7B9100AE181

Fig. 3A–C

#### Type material examined.

***Holotype***: Korea • 1♂ (slide no. NIBRIN0000941947); Gangwon-do, Pyeongchang-gun, Jinbu-myeon, Odaesan-ro, Mt. Odae, small valley before So-Myeong valley **(Odae 1)**; 18 Apr. – 1 May 2020; D. Ham, S. Park leg.; deposited in NIBR.

#### Diagnosis.

**Figure 3.**
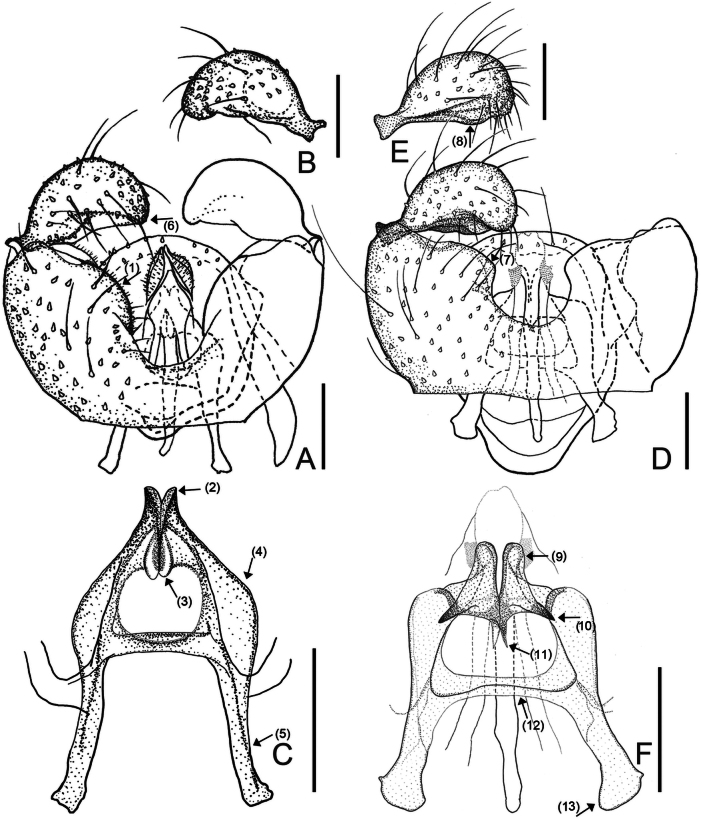
Male morphology of *Campylomyzaangusta* sp. nov., holotype **A–C** and *Campylomyzaconvexa* sp. nov., holotype **D–F**: **A** gonocoxites, ventral veiw **B** gonostylus, dorsal view **C** tegmen, dorsal view **D** gonocoxites, ventral veiw **E** gonostylus, dorsal view **F** tegmen, dorsal view. Scale bars: 0.05 mm.

*Campylomyzaangusta* sp. nov. belongs to the *ormerodi* group of species where it is reminiscent of *C.pubescens* (Jaschhof and Jaschhof 2009) mainly due to cerci bearing strikingly large pubescence and short dorsal transverse bridge, which is almost not protruding beyond the ventrobasal margin. *Campylomyzaangusta* sp. nov. is distinguished as follows. Gonostyli moderately convex posteriorly with narrowly rounded apex (Fig. 3A, ↓_6_). Gonocoxites strongly protruding dorsomedially, ventral bridge short. The tegmen lacks shoulders (Fig. 3C, ↓_4_), parameral apodemes long (Fig. 3C, ↓_5_), apical points directed slightly laterally, mesal points short, weakly sclerotized, rounded apically (Fig. 3C, ↓_3_).

#### Measurements.

Male adult (holotype): Body length 1.329 mm. Wing length 1.569 mm. Hind leg coxa 0.160 mm; femur 0.571 mm; tibia 0.567 mm; tarsomere I 0.330 mm; tarsomere II 0.153 mm; tarsomere III 0.109 mm; tarsomere IV 0.078 mm; tarsomere V 0.061 mm.

#### Description.

Male adult (holotype). ***Head*.** Postocular bristles seven. Antenna with 12 flagellomeres. Neck of fourth antennal flagellomeres shorter than node. Node with one complete and two incomplete crenulate whorls with sensory hairs, two incompletely collar-shaped sensilla distally. Palpus 4-segmented; fourth segment longest. ***Thorax*.** Preepisternum with eight setae. Wing length to width ratio 2.53. AntC ending beyond R_4+5_ but before reaching M_4_; ApicR_1_ 4.18× length of Rs. CuA separated. Tarsomere I longer than tarsomere II. Claws sickle-shaped, fine toothed; empodia shorter than claws; pubescent. ***Terminalia*.** Tg9 tapered towards apex with 13 setae apically. Ventral bridge of gonocoxites short, dorsal transverse bridge protruding only slightly beyond the ventrobasal margin. Ventral emargination deep, U-shaped. Ventromedial portion broad rounded, not protruding medially (Fig. 3A, ↓_1_). Gonostyli rounded apically with semi-circular apical margin; excavated ventromedially, plump dorsally; setae denser towards apex. On tegmen, apical points not lamellate, pointed apically, directed slightly posterolaterally (Fig. 3C, ↓_2_); mesal points weakly sclerotized, short, tapered basally, broadened at apical third, rounded apically (Fig. 3C, ↓_3_). Tegmen shoulders indistinct (Fig. 3C, ↓_4_). Parameral apodemes long, more than half-length of tegmen (Fig. 3C, ↓_5_).

#### Etymology.

The specific epithet *angusta* in Latin means narrow, referring to the narrowness of the shoulder region of the tegmen in this species.

### ﻿ Campylomyza cavitata

Taxon classificationAnimaliaDipteraCecidomyiidae

Mamaev, 1998

AD6365B2-9267-5E2A-B330-779926A0ACEC

Campylomyza cavitata Mamaev, 1998: 7; Jaschhof and Jaschhof 2009: 112–113, fig. 37A–C.

#### Specimens examined.

Korea • 1♂ (slide no. NIBR0000919409); **Odae 1**; 11–26 May 2019; D. Ham, S. Park leg.; deposited in NIBR.

#### Distribution.

Sweden, Finland, Germany, Russia, new record for South Korea.

### ﻿ Campylomyza cingulata

Taxon classificationAnimaliaDipteraCecidomyiidae

Jaschhof, 2009

CB1B5D20-6DA2-5D84-B9D4-EBA6F558C1CB

Campylomyza cingulata Jaschhof, 2009: 119, fig. 41A–E.

#### Specimens examined.

Korea • 1♂ (slide no. NIBRIN0000941946); **Odae 1**; 18 Apr. – 1 May 2020; D. Ham, S. Park leg.; deposited in NIBR.

#### Distribution.

Fennoscandia, Germany, new record for South Korea.

### ﻿ Campylomyza convexa sp. nov.

Taxon classificationAnimaliaDipteraCecidomyiidae

52FC3E3F-8F3D-5173-9C8D-1A6378DC38C6

https://zoobank.org/2A011D80-C1EE-4EB4-BCB1-787C972052F8

Fig. 3D–F

#### Type material examined.

Korea • 1♂ (slide no. HDS-505); Gyeonggi-do, Namyangju-si, Wabu-eup, Dosim-gil, Korea University’s farm to practice **(KUF)**; 2–8 Apr. 2017; D. Ham leg.; deposited in KU. ***Paratypes***: Korea • 1♂ (slide no. HDS-504); same data and deposition as holotype • 2♂♂ (slides no. NIBRIN0000857555, NIBRIN0000919405) **KUF**; 2–8 Apr. 2017; D. Ham leg.; deposited in NIBR.

#### Other material examined.

Korea • 4♂♂ (slides no. NIBRIN0000992649 – NIBRIN0000992652); **KUF**; 2–8 Apr. 2017; Y. J. Bae leg.; deposited in NIBR.

#### Diagnosis.

*Campylomyzaconvexa* sp. nov. is most similar to *C.aemula* (cf. Jaschhof and Jaschhof 2009: fig. 29A–D), especially in having the rounded apical points on the tegmen, tapering posteriorly, the dorsal processes are broad basally, directed dorsolaterally with a strongly sclerotized triangular apex. However, *C.convexa* sp. nov. can be distinguished from *C.aemula* by following characteristics: 1) Gonostyli moderately convex apically, not excavated medially, broadly rounded apically, with small dorsomedial lobe (Fig. 3E, ↓_8_); 2) Apical points of tegmen parallel-sided to rounded apically, longer than *C.aemula* (Jaschhof and Jaschhof 2009: 102); 3) Mesal points of tegmen longer and narrower than in *C.aemula* (Fig. 3E, ↓_11_); 4) Dorsal processes lacking sclerotized ridge, strongly sclerotized apically (Fig. 3E, ↓_10_).

#### Measurements.

Male adult (holotype): Body length 1.417 mm. Wing length 1.639 mm. Hind leg coxa 0.105 mm; femur 0.555 mm; tibia 0.574 mm; tarsomere I 0.333 mm; tarsomere II 0.170 mm; tarsomere III 0.138 mm; tarsomere IV 0.082 mm; tarsomere V 0.065 mm.

#### Description.

Male adult (holotype). ***Head*.** Postocular bristles seven. Antenna with 12 flagellomeres. Neck of fourth antennal flagellomeres shorter than node. Node with one complete and two incomplete crenulate whorls with sensory hairs, two incompletely collar-shaped sensilla distally. Palpus 4-segmented; fourth segment longest. ***Thorax*.** Preepisternum with nine setae. Wing length to width ratio 2.24. AntC ending beyond R_4+5_ but before reaching M_4_; ApicR_1_ 3.82× length of Rs. CuA separated. Tarsomere I longer than tarsomere II. Tarsomere I longer than tarsomere II. Claws sickle-shaped, slightly toothed; empodia as long as claws, slightly broaden apically; pubescent. ***Terminalia*.** Tg9 tapered towards apex with eight fine setae. Ventral bridge of gonocoxites long, ventral emargination relatively short and broad, U-shaped, dorsal transverse bridge broad, extending far beyond ventrobasal margin. Ventromedial portion of gonocoxites broad, slightly pronounced (Fig. 3D, ↓_7_). Gonostyli curved anteroventrally, rounded apically, moderately convex apically with small dorsomedial lobe (Fig. 3E, ↓_8_); setae distributed evenly in ventral view, denser towards apex in dorsal view. On tegmen, apical points long, parallel-sided to rounded apically, not lamellate, sclerotized (Fig. 3F, ↓_9_); dorsal processes strongly sclerotized apically, directed dorsolaterally (Fig. 3F, ↓_10_); mesal points weakly sclerotized, faint apically, directed anteriorly (Fig. 3F, ↓_11_). Shoulders of tegmen inconspicuous. Transverse brace rib-shaped without extension (Fig. 3F, ↓_12_). Parameral apodeme sclerotized, long, slightly shorter than half of tegmen (Fig. 3F, ↓_13_). Ejaculatory apodeme of typical *Campylomyza* outline.

#### Etymology.

From the Latin word *convexus*, meaning ‘a surface with rounded edges’, which refers to the rounded outline of the apex of the apical points on the tegmen.

### ﻿ Campylomyza cornigera sp. nov.

Taxon classificationAnimaliaDipteraCecidomyiidae

451E00DA-591F-584F-85A2-41FC1F6834CC

https://zoobank.org/52110DC2-AC64-4EAC-AC9A-639CBFD1C1AF

Fig. 4A–C

#### Type material examined.

***Holotype***: Korea • 1♂ (slide no. 19Aya-8); Gyeongsangbuk-do, Yeongyang-gun, Yeongyang-eup, Gowol-gil, 23, National Endangered Species Restoration Center **(NERC)**; 10–17 Apr. 2019; Y. J. Choi, H. G. Kim leg.; deposited in KU. ***Paratype***: Korea • 1♂ (slide no. NIBRIN0000857558); **KUF**; 2–8 Apr. 2017; D. Ham leg.; deposited in NIBR.

#### Other material examined.

Korea • 1♂ (slide no. NIBRIN0000992637); **Odae 1**; 18 Apr. – 1 May 2020; D. Ham, S. Park leg.; deposited in NIBR.

#### Diagnosis.

**Figure 4.**
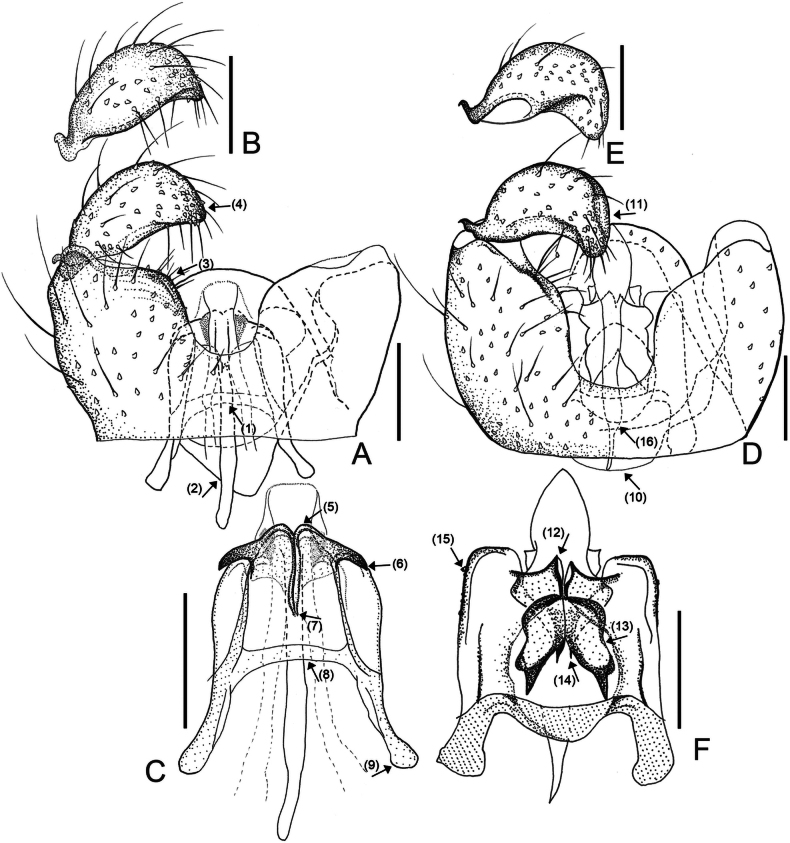
Male morphology of *Campylomyzacornigera* sp. nov., holotype **A–C** and *Campylomyzahori* sp. nov., holotype **D–F**: **A** gonocoxites, ventral view **B** gonostylus, dorsal view **C** tegmen, dorsal view **D** gonocoxites, ventral view **E** gonostylus, dorsal view **F** tegmen, dorsal view. Scale bars: 0.05 mm.

*Campylomyzacornigera* sp. nov. is most similar to *C.nigroliminata* Mamaev, 1998 (cf. Jaschhof and Jaschhof 2021: fig. 30A, B), especially in having lamellate apical points of the tegmen that are rounded apically and pointed processes directed anterolaterad (Fis. 4C, ↓_5, 6_), and mesal processes are directed anteriorly (Fig. 4C, ↓_7_). However, *C.cornigera* sp. nov. can be distinguished from *C.nigroliminata* by the following characteristics: 1) Pointed processes directed anterolaterally of apical points slightly curved; 2) Dorsal processes missing; 3) Tegmen shoulders indistinct.

#### Measurements.

Male adult (holotype): Body length 1.315 mm. Wing length 1.574 mm. Hind leg coxa 0.141 mm; femur 0.539 mm; tibia 0.515 mm; tarsomere I 0.302 mm; tarsomere II 0.142 mm; tarsomere III 0.105 mm; tarsomere IV 0.062 mm; tarsomere V 0.056 mm.

#### Description.

Male adult (holotype). ***Head*.** Postocular bristles seven. Antenna with 12 flagellomeres. Neck of fourth antennal flagellomeres as long as node. Node with one complete and two incomplete crenulate whorls with sensory hairs, two incompletely collar-shaped sensilla distally. Palpus 4-segmented; fourth segment longest. ***Thorax*.** Preepisternum with 1–9 setae. Wing length to width ratio 2.58. AntC ending beyond R_4+5_ but before reaching M_4_; ApicR_1_ 3.08× length of Rs. CuA separated. Tarsomere I longer than tarsomere II. Claws sickle-shaped, toothed; empodia longer than claws, slightly broaden apically; pubescent. ***Terminalia*.** Tg9 tapered towards apex with seven or eight fine setae apically. Ventral bridge of gonocoxites long (Fig. 4A, ↓_1_), with U-shaped emargination; dorsal transverse bridge narrowly tapering, extending far beyond basal margin (Fig. 4A, ↓_2_). Ventromedial portion of gonocoxites almost angular (Fig. 4A, ↓_3_). Gonostyli narrowly rounded to pointed apically (Fig. 4A, ↓_4_), moderately convex posteriorly, and slightly excavated medially, plump dorsally; setae denser towards apex. Tegmen long and narrow, apical points sclerotized, short, stout, and broadly rounded apically (Fig. 4C, ↓_5_); a pair of strongly sclerotized processes directed dorsolaterally (Fig. 4C, ↓_6_); true dorsal processes missing. Mesal points of tegmen slightly sclerotized, narrowly long, directed anteriorly (Fig. 4C, ↓_7_). Shoulders of tegmen inconspicuous; width between apices of shoulders narrower than processes directed dorsolaterally. Transverse brace rib-shaped (Fig. 4C, ↓_8_). Parameral apodemes long (Fig. 4C, ↓_9_). Ejaculatory apodeme of typical *Campylomyza* outline.

#### Etymology.

The species epithet cornigera, derived from Latin meaning ‘having horns,’ refers to the horn-shaped processes on the tegmen that are directed dorsolaterally.

### ﻿ Campylomyza cornuta

Taxon classificationAnimaliaDipteraCecidomyiidae

Jaschhof, 1998

17378D71-DA3D-5C7A-8255-B768107F116B

Campylomyza cornuta Jaschhof 1998b: 260–261, Abb. 1a–e.

#### Specimens examined.

Korea • 2♂♂ (slides no. NIBRIN0000941945, NIBRIN0000992653); **HN**; 3 Mar. – 12 Apr. 2019; H. S. Ahn leg.; deposited in NIBR.

#### Distribution.

Sweden, Lithuania, Germany, and new to South Korea.

### ﻿ Campylomyza hori sp. nov.

Taxon classificationAnimaliaDipteraCecidomyiidae

3668EB2B-E84E-53D9-89C6-7C09FC656247

https://zoobank.org/8D43A3F5-2FF5-4E08-BDD6-85A776E46646

Fig. 4D–F

#### Type material examined.

***Holotype***: Korea • 1♂ (slide no. 19I-5); Gangwon-do, Jeongseon-gun, Jeongseon-eup, Hoedong-ri, 870, Gariwangsan Recreational Forest **(Gariwang)**; 13 Apr. – 12 May 2019; D. Ham, S. Park leg.; deposited in KU. ***Paratype***: Korea • 1♂ (slide no. NIBRIN0000919401); same data as holotype and deposited in NIBR.

#### Other material examined.

Korea • 2♂♂ (slides no. NIBRIN0000992654, NIBRIN0000992655); **Sobaek**; 6 May – 6 Jun. 2019; D. Ham, S. Park leg.; deposited in NIBR.

#### Diagnosis.

*Campylomyzahori* sp. nov. is most similar to *C.mohrigi* Jaschhof, 2009, especially in having the apical points divided, and the dorsal processes with sclerotized ridge, subtriangular apex on tegmen. However, *C.hori* sp. nov. can be distinguished from *C.mohrigi* by the following characteristics: 1) Necks of antennal flagellomeres longer than nodes; 2) Gonostyli slightly longer and narrower (Fig. 4E); 3) Dorsal processes wider, margin sclerotized, with subtriangular apex, center membranous (Fig. 4F, ↓_13_) vs. narrower, leaf-shaped with sclerotized ridge and points apically in *C.mohrigi*.

#### Measurements.

Male adult (holotype). Body length 1.441 mm, wing length 1.645 mm. Hind leg coxa 0.156 mm; femur 0.607 mm; tibia 0.647 mm; tarsomere I 0.351 mm; tarsomere II 0.180 mm; tarsomere III 0.141 mm; tarsomere IV 0.088 mm; tarsomere V 0.070 mm.

#### Description.

Male adult (holotype). ***Head*.** Postocular bristles three. Antenna with 12 flagellomeres. Neck of fourth antennal flagellomere longer than node. Node with one complete and two incomplete crenulate whorls with sensory hairs, two incompletely collar-shaped sensilla distally. Palpus 4-segmented; fourth segment longest. **Thorax.** Preepisternum with five fine setae anteriorly. Wing length to width ratio 2.47. AntC ending beyond R_4+5_ but before reaching; ApicR_1_ 2.77× length of Rs. CuA separated. Tarsomere I longer than tarsomere II. Claws sickle-shaped, weakly toothed; empodia as long as claws; pubescent. ***Terminalia*.** Tg9 tapering towards apex with six fine setae apically. Ventral emargination deep, U-shaped, ventral bridge short. Dorsal transverse bridge broadly rounded apically, slightly extended beyond ventrobasal margin (Fig. 4D, ↓_10_). Gonostyli elongated apically, curved anteroventrally, constricted ventrosubapically (Fig. 4D, ↓_11_) with fine setae denser towards apex; incised dorsomesally. On tegmen, apical points pointed, directed posteriorly (Fig. 4F, ↓_12_); dorsal processes broad basally, constricted medially, pointed apically (Fig. 4F, ↓_13_), directed anterodorsally, with strongly sclerotized margin basally; mesal points faint, short, pointed (Fig. 4F, ↓_14_). Tegmen shoulders almost angular, equipped with several small bumps laterally (Fig. 4F, ↓_15_). Transverse brace with lobe-like dorsal extensions. Ejaculatory apodeme swelling medially (Fig. 4D, ↓_16_), narrow basally.

#### Etymology.

The species epithet *hori* originates from the Korean native term, pronounced ‘hori-hori-hada’, an adjective describing a slender or tapered part. This name specifically denotes the narrowed part of the gonostyli.

### ﻿ Campylomyza odae sp. nov.

Taxon classificationAnimaliaDipteraCecidomyiidae

FB7F0A26-6D62-5ECE-BE3E-C29C56C9E0FF

https://zoobank.org/4901DF68-5CC0-4023-A8C6-5D29649014E0

Fig. 5A–D

#### Type material examined.

***Holotype***: Korea • 1♂ (slide no. NIBRIN0000992641) Gangwon-do, Pyeongchang-gun, Jinbu-myeon, Odaesan-ro, Mt. Odae, the road before temple (Buk-Dae-Mi-Reuk-Am) **(Odae 2)**; 11–26 May 2019; D. Ham, S. Park leg.; deposited in NIBR. ***Paratype***: Korea • 1♂ (slide no. NIBRIN0000919408); same data and deposition as holotype.

#### Diagnosis.

**Figure 5.**
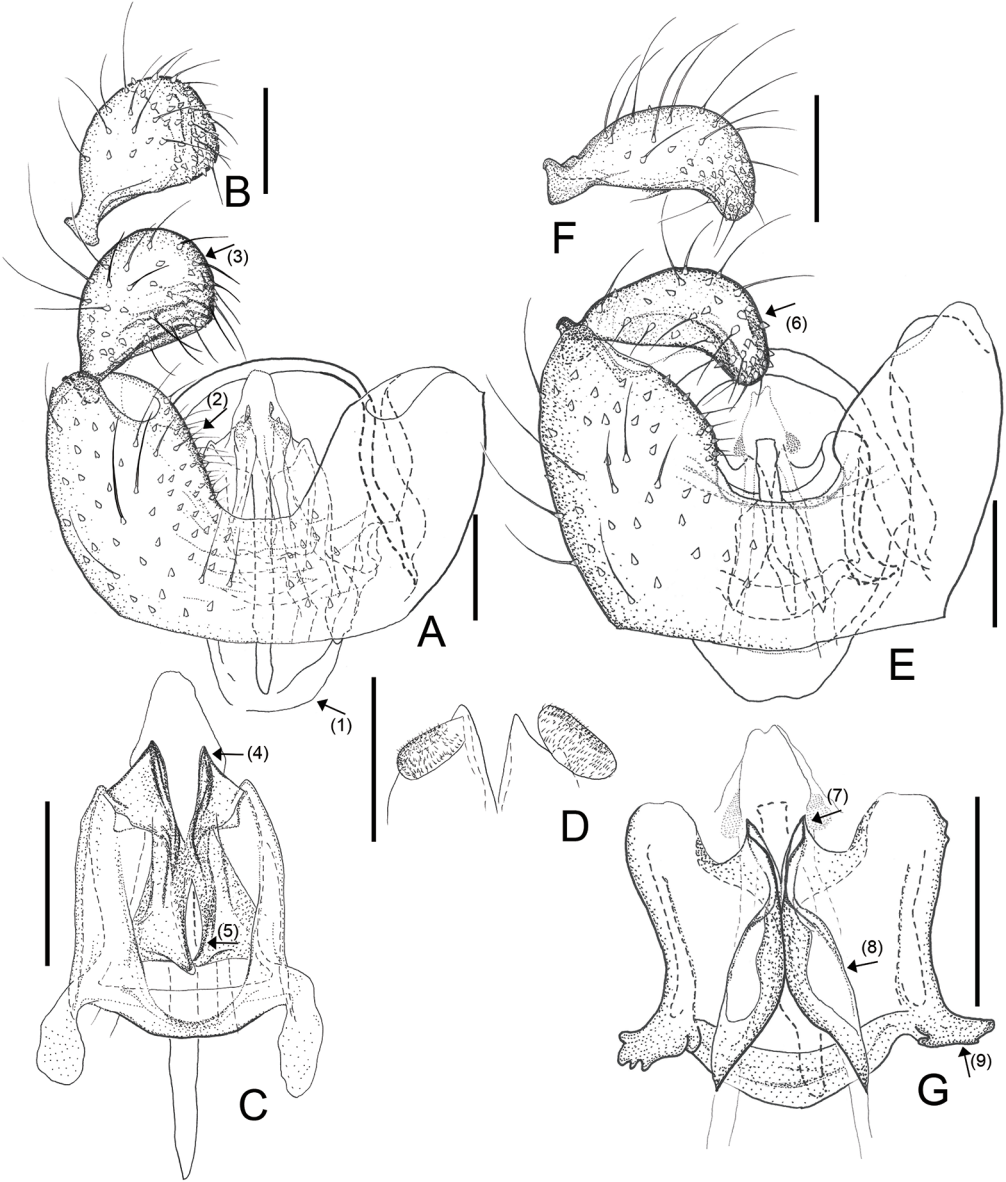
Male morphology of *Campylomyzaodae* sp. nov., holotype **A–D** and *Campylomyzasalicia* sp. nov., holotype **E–G**: **A** gonocoxites, ventral view **B** gonostylus, dorsal view **C** tegmen, dorsal view **D** cerci, ventral view **E** gonocoxites, ventral view **F** gonostylus, dorsal view **G** tegmen, dorsal view. Scale bar: 0.05 mm.

*Campylomyzaodae* sp. nov. is distinguishable from other *Campylomyza* species by the following combination of characteristics: 1) Apical margin of gonostyli rounded (Fig. 5A, ↓_3_); 2) Dorsal processes on tegmen with inconspicuous pair of subtriangular processes anterolaterally; 3) Dorsal processes constricted medially, forming mesal cleft (Fig. 5C, ↓_6_); 4) Cerci visible (Fig. 5D).

#### Measurements.

Male adult (Holotype): Body length 1.737 mm. Wing length 1.870 mm. Hind leg coxa 0.221 mm; femur 0.713 mm; tibia 0.624 mm; tarsomere I 0.369 mm; tarsomere II 0.193 mm; tarsomere III 0.134 mm; tarsomere IV 0.089 mm; tarsomere V 0.072 mm.

#### Description.

Male adult. Slightly larger than other *Campylomyza* species. ***Head*.** Postocular bristles four. Antenna with 12 flagellomeres. Neck of fourth antennal flagellomere slightly shorter than node. Node with one complete and two incomplete crenulate whorls with sensory hairs, two incompletely collar-shaped sensilla distally. Palpus 4-segmented; fourth segment longest. ***Thorax*.** Wing length to width ratio 2.39, AntC ending beyond R_4+5_ but before reaching M_4_; ApicR_1_ 4.31× length of Rs; CuA separated. Tarsomere I longer than tarsomere II. Claws sickle-shaped, toothed; empodia small, shorter than claws. ***Terminalia*.** Tg9 broadly tapered towards apex with eight fine setae. Ventral emargiantion U-shaped; ventral bridge long; dorsal transverse bridge extending far beyond ventrobasal margin (Fig. 5A, ↓_1_); ventromedial portion of gonocoxites relatively narrow, not pronounced (Fig. 5A, ↓_2_). Gonostyli short, stout, strongly convex posteriorly, truncated apically (Fig. 5A, ↓_3_), directed ventrally, bearing dorsoapically numerous straight setae of various length, with denser stiff setae as it goes to apex. Tegmen narrow, shoulders inconspicuous. Parameral apodemes short. Apical points lamellated, triangular-shaped, directed posteriorly (Fig. 5C, ↓_4_), separated by wide cleft mesally; dorsal processes subtriangular shaped, directed dorsomedially, apices crossed with spreading subtriangular extensions (Fig. 5C, ↓_5_); each dorsal process constricted at midlength, forming mesal cleft (Fig. 5C, ↓_6_). Cerci visible.

#### Etymology.

The species name *odae* is a noun in apposition to the collection locality, Mt. Odae in Gangwon province.

### ﻿ Campylomyza salicia sp. nov.

Taxon classificationAnimaliaDipteraCecidomyiidae

471DC710-F359-5A73-8758-32F3C4581E23

https://zoobank.org/53F03A9D-EBE9-4D6A-9599-65617BADF8FC

Fig. 5E–G

#### Type material examined.

***Holotype***: Korea • 1♂ (slide no.19Aya-2); Gyeongsangbuk-do, Yeongyang-gun, Yeongyang-eup, Gowol-gil, 23, National Endangered Species Restoration Center **(NERC)**; 10–17 Apr. 2019; Y. J. Choi, H. G. Kim leg.; deposited in KU. **Paratypes**: Korea • 5♂♂ (slides no. 19AY-5, 10, 13, 17, 19AYa-13); same data and deposition as holotype • 1♂ (slide no. NIBRIN0000919404); same data as holotype, deposited in NIBR.

#### Other material examined.

Korea • 2♂♂ (slides no. 19AZ-6, 9); **NERC**; 3–10 Apr. 2019; Y. J. Choi, H. G. Kim leg.; deposited in KU • 1♂ (slide no. 19AX-4); **NERC**; 20–27 Mar. 2019; Y. J. Choi, H. G. Kim leg.; deposited in KU • 1♂ (slide no. NIBRIN0000992640); **NERC**; 27 Mar. – 3 Apr. 2019; Y. J. Choi, H. G. Kim leg.; deposited in NIBR.

#### Diagnosis.

*Campylomyzasalicia* sp. nov. is most similar to *C.mohrigi* (cf. illustration of Jaschhof and Jaschhof 2009: 109), especially having the elongated, tapering Gonostyli ventrally, not lamellate apical points and foliate dorsal processes which reaching to transverse brace on tegmen. However, *C.salicia* sp. nov. can be distinguished from *C.mohrigi* by following characteristics: 1) dorsal processes of tegmen sclerotized margin without sclerotized ridge (Fig. 5G, ↓_9_); 2) the dorsal processes are directed dorsolaterally with a strongly sclerotized triangular apex; 3) parameral apodeme shorter than that of *C.mohrigi* (Fig. 5G, ↓_10_).

#### Measurements.

Male adult (holotype): Body length 0.944 mm. Wing length 1.266 mm. Hind leg coxa 0.142 mm; femur 0.441 mm; tibia 0.463 mm; tarsomere I 0.275 mm; tarsomere II 0.123 mm; tarsomere III 0.103 mm; tarsomere IV 0.072 mm; tarsomere V 0.057 mm.

#### Description.

Male adult (holotype). Slightly smaller than other *Campylomyza* species. ***Head*.** Postocular bristles five. Antenna with 12 flagellomeres. Neck of fourth antennal flagellomere longer than node. Node with one complete and two incomplete crenulate whorls with sensory hairs, two incompletely collar-shaped sensilla distally. Palpus 4-segmented; fourth segment longest. ***Thorax*.** Preepisternum with six fine setae anteriorly. Wing length to width ratio 2.70. AntC ending beyond R_4+5_ but before reaching M_4_; ApicR_1_ 3.23× length of Rs; CuA separated. Tarsomere I longer than tarsomere II. Claws slightly toothed; empodia small, narrow. ***Terminalia*.** Tg9 tapered towards apex with 8 setae apically. Ventral bridge of gonocoxites half-length of gonocoxites; dorsal transverse bridge tapering, extending beyond ventrobasal margin. Ventral emargination U-shaped. Gonostyli elongated, blunt to slightly pointed apically, moderately convex apically; ventrosubapically constricted (Fig. 5E, ↓_7_); excavated ventromesally; setae denser towards apex. On tegmen, apical points triangular shaped, not lamellate, pointed apically, directed posterolaterally (Fig. 5G, ↓_8_); dorsal processes leaf-shaped, elongated slightly beyond transverse brace, with strongly sclerotized apex (Fig. 5G, ↓_9_). Shoulders of tegmen well developed, thick. Transverse brace slightly extended, lobe shaped. Parameral apodemes short (Fig. 5G, ↓_10_).

#### Etymology.

The species name *salicia* is derived from the Latin word *salici*, meaning ‘willow,’ in reference to the dorsal processes of the tegmen, which resembles the shape of a willow leaf.

##### ﻿Key to the Species of Korean *Campylomyza*

**Table:** 

1	Ejaculatory apodeme without apical extension. Tegmen without dorsal processes	***C.cornuta* Jaschhof, 1998**
–	Ejaculatory apodeme with apical extension. Tegmen bearing apical points and processes	**2**
2	On tegmen, dorsal processes forming mesal cleft with expended subtriangular processes laterally	***C.odae* sp. nov. (Fig. 5A–D)**
–	On tegmen, dorsal processes elongated without mesal cleft or absent, or dorsomesal processes present	**3**
3	On tegmen, apical points present; dorsal processes elongated, foliate or dorsolaterad processes horn-shaped at apex; shoulders well developed	***flavipes* group, 4**
–	On tegmen, dorsal processes absent or single pair of small dorsomesal processes present	**12**
4	Gonostyli short, ovoid-shaped; not pointed	***C.abjecta* Mamaev, 1998**
–	Gonostyli elongated, curved inwardly	**5**
5	Gonostyli elongated, strongly curved inwardly; constricted ventrosubapically with dense setae apically. On tegmen, apical points sharp apically	**6**
–	Gonostyli blunt, rounded apically; slightly curved inwardly	**7**
6	Ventral bridge of gonocoxites shorter than half-length of gonocoxites. Dorsal processes wide, constricted at middle length with sclerotized subtriangular points on tegmen	***C.hori* sp. nov. (Fig. 4D–F)**
–	Ventral bridge of gonocoxites half-length of gonocoxites. Dorsal processes tapering sclerotized along margin, beyond transverse brace. Parameral apodeme short	***C.salicia* sp. nov. (Fig. 5A–D)**
7	On tegmen, apical points rounded apically, parallel-sided	**8**
–	On tegmen, apical points pointed at apex (apical points of *C.ambulata* typically pointed, subtriangular, but occasionally bulged with round serrated surfaces)	**10**
8	Gonocoxites with swollen, pronounced ventromedial portion. On tegmen, dorsal processes spoon-shaped; elongated with subtriangular narrow tips apically, directed anteriorly	***C.aborigena* Mamaev, 1998 (Fig. 2A–C)**
–	On tegmen, dorsal processes horn-shaped, sclerotized apically, directed laterally	**9**
9	On tegmen, apical points rounded, short, slightly curved anterolaterally	***C.cornigera* sp. nov. (Fig. 4A–C)**
–	Gonostyli with small dorsomedial lobe. On tegmen, apical points long, parallel-sided to rounded apically; dorsal processes strongly sclerotized, straightened, directed dorsolaterally with triangular apex	***C.convexa* sp. nov. (Fig. 3D–F)**
10	On tegmen, apical points pointed or broadly rounded apically with serrated surfaces; dorsal processes elongated, directed anteriorly	***C.ambulata* sp. nov. (Fig. 2D–G)**
–	On tegmen, dorsal processes with subtriangular apex, directed anterolaterally	**11**
11	Gonostyli slightly excavated mesally, flattened dorsally, narrowly rounded apically with convex apical margins; Tegmen apical points lamellate; dorsal processes foliate, directed anterolaterally	***C.flavipes* Meigen, 1818**
–	Gonostyli neither curved nor excavated; tegmen apical points lamellated with dorsal processes elongated and foliate; sclerotized along margins and directed anterolaterally or anteriorly	***C.furva* Edwards, 1938**
12	On tegmen, apical points tapering; dorsal processes absent; mesal processes rounded at apex; shoulders indistinct or missing	***ormerodi* group, 13**
–	Tegmen with 1 pair of small dorsomesal processes which are serrated or extended	**14**
13	Tg9 tapering broadly. Cerci with strikingly large pubescence. Tegmen shoulders inconspicuous; parameral apodeme long, more than half-length of tegmen	***C.angusta* sp. nov. (Fig. 3A–C)**
–	Tg9 tapering narrowly. Gonocoxites with protruding portion dorsomesally. On tegmen, apical points slightly serrated; shoulder indistinct. Parameral apodeme short	***C.cavitata* Mamaev, 1998**
14	Tegmen processes serrated	***serrata* group, 15**
–	On tegmen, apical points rounded on apex in ventral view; medial structure strongly sclerotized, connected with triangular structure below	***C.cingulata* Jaschhof, 2009**
15	Gonostyli with lobe dorsally. Serrated processes on tegmen situated subapically dorsomedially in longitudinal direction. Aedeagal head small	***C.appendiculata* Jaschhof, 2015**
–	Medial bridge of gonocoxites with one spine, one pubescent projection. Serrate processes on tegmen turned posteriorly forming apex of tegmen. Aedeagal head large	***C.spinata* Jashchhof, 1998**

##### ﻿DNA barcode analysis and MOTU estimation

**Figure 6.**
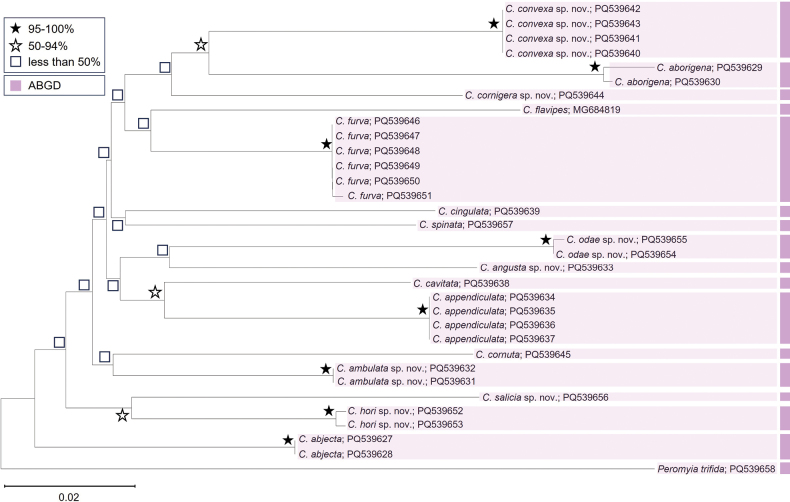
Neighbor-joining (NJ) Kimura-2-parameter tree derived from the *COI* analysis of sixteen Korean *Campylomyza* species, with *Peromyiatrifida* as the outgroup. Numbers at the nodes represent NJ bootstrap support values. The vertical purple color bar on the right represents results from the ABGD delimitation method.

The 658-bp *COI* sequences were analyzed, revealing 234 variable sites, of which 63 were parsimony informative. Within the genus *Campylomyza*, the interspecific divergences (*p*-distances) ranged from 6.18% to 15.28%. The mean distance across the entire dataset was 10.72% (Suppl. material 1: table S3). Intraspecific genetic distances varied from 0% to 0.92%. The species delimitation using the ABGD method delineated 17 Molecular Operational Taxonomic Units (MOTUs), including the outgroup. These MOTUs are illustrated by the color bars on the Neighbor Joining (NJ) tree in Fig. 6, corresponding with the delineations observed in the same NJ tree. All 17 MOTUs correspond to groups distinguished by their morphological characteristics.

## ﻿Discussion

The discovery of *C.abjecta* and *C.aborigena* in Korea is particularly significant due to the inherent challenges in studying fungivorous cecidomyiids. This finding underscores the need to expand research on species previously known only from single specimens in Russia. Initially, the type specimens of these species were based on solitary samples that lacked adequate descriptions and illustrations, limiting precise identification. Given the geographic proximity between Korea and Russia, it is crucial to broaden research efforts on these species. In 2006, Dr. Mathias Jaschhof visited the Russian Zoological Museum and sketched the type specimens, which greatly aided in their identification in Korea (M. Jaschhof pers. comm. Oct. 2019). Notably, *C.abjecta* has also been recorded in Europe, particularly in Sweden (Jaschhof and Jaschhof 2017), while the finding of *C.aborigena* in Korea marks only the second confirmed sighting since its original description in Russia. Considering Russia’s vast size, the presence of these species in both European and Far Eastern regions complicates our understanding of their biogeography. This situation highlights the importance of thoroughly studying all previously described species, as some, like those discussed here, may be more widespread than previously thought. The discovery of *C.aborigena* in Korea is especially significant because it not only confirms the species’ presence in a new region but also emphasizes its ecological and taxonomic relevance, contributing to a more comprehensive understanding of this group.

This study extends the application of DNA barcoding for species delimitation within the genus *Campylomyza*, beyond the initial barcoding of individual species such as *C.flavipes* for subfamily relationship analysis within the Cecidomyiidae (Sikora et al. 2019). Comprehensive mitochondrial Cytochrome Oxidase subunit I (mtCOI) data is provided for all 16 documented species, encompassing seven that are newly described and five that are newly reported in Korea. The analyses of the Neighbor Joining (NJ) tree and genetic divergence have effectively differentiated all species by their interspecific variations, with interspecific divergences noted to be between 6.18% and 15.28% (Suppl. material 1: table S3). The maximum intraspecific genetic distance (0.92%) was significantly smaller than the minimum interspecific one (6.18%). The NJ tree (Fig. 6) strongly supported the monophyly of each new species and the overall monophyly of the genus *Campylomyza*. Moreover, the species identifications are in agreement with the Automated Barcode Gap Discovery (ABGD) results, as depicted in Fig. 6.

The genus *Campylomyza* encompasses a diverse range of species occurring across different continents, including the Holarctic, Neotropical, Oriental, and Australasian/Oceanian regions (Gagné and Jaschhof 2021). *Campylomyza* are characterized by their small size, typically measuring around 1.0–1.8 mm and exhibit distinctive behaviors such as clustering and swarming during the mating season, which typically occurring in cooler weather such as April or November (Kanmiya and Yukawa 2020). Our study utilized Malaise traps to collect this aggregation and provide insights into their geographical distribution and species occurrence patterns. Notably, some species occur in large numbers in the *Campylomyza* population, but only a few individuals of some species can be identified. For example, *C.convexa*, *C.appendiculata*, and *C.furva* occurred collectively, but only a small number of individuals of *C.odae* and *C.angusta* were found to have occurred. In addition, our findings revealed instances of sympatric occurrence, where multiple species coexisted within the same sampling sites. In the National Endangered Species Restoration Center in Yeongyang, (Table 1, Suppl. material 1: table S1) region, we observed up to eight species occurring together at a single location, highlighting the coexistence and potential ecological interactions among these species. Furthermore, our investigations unveiled cases of wide distribution for certain species. For instance, *C.abjecta* was found in Namyangju in Gyeonggi-do, as well as Yeongyang and Sangju in Gyeongsangbuk-do. *Campylomyzacornigera* sp. nov. was documented in Namyangju in Gyeonggi-do, Yeongyang and Pyeongchang in Gangwon-do (Table 1, Suppl. material 1: table S1). These finding emphasize the complexity of species assemblages within the genus *Campylomyza* and shed light on their ecological dynamics. By studying the microhabitat preferences and geographic distributions, we contribute to the understanding of species diversity and their spatial patterns. Such knowledge is crucial for comprehensive biodiversity assessments and conservation efforts.

## Supplementary Material

XML Treatment for Campylomyza

XML Treatment for Campylomyza abjecta

XML Treatment for Campylomyza aborigena

XML Treatment for Campylomyza ambulata

XML Treatment for Campylomyza angusta

XML Treatment for Campylomyza cavitata

XML Treatment for Campylomyza cingulata

XML Treatment for Campylomyza convexa

XML Treatment for Campylomyza cornigera

XML Treatment for Campylomyza cornuta

XML Treatment for Campylomyza hori

XML Treatment for Campylomyza odae

XML Treatment for Campylomyza salicia

## ﻿Additional information

### Author ORCIDs

Daseul Ham https://orcid.org/0000-0002-1900-3637

Yeon Jae Bae https://orcid.org/0000-0001-7810-5409

### Data availability

All of the data that support the findings of this study are available in the main text or Supplementary Information.

## Supplementary materials

Supplementary material 1 Additional information Data type xlsx Explanation note **table S1.** Detailed location data for collected *Campylomyza* species in Korea and species occurrence by region; **table S2.** Accession numbers of *Campylomyza* species deposited in NIBR and NCBI for *COI* sequence data; **table S3.** The percentage of genetic divergence between sequences was estimated, and the number of base differences per site between sequences is presented (Cells with values below 3% are shaded in gray; important values are highlighted in red). Standard errors, expressed as percentages, are provided above the diagonal and were calculated using a bootstrap procedure with 1,000 replicates. Positions with gaps and missing data were excluded from the analysis (complete delete option). https://binary.pensoft.net/file/1216610This dataset is made available under the Open Database License (http://opendatacommons.org/licenses/odbl/1.0/). The Open Database License (ODbL) is a license agreement intended to allow users to freely share, modify, and use this Dataset while maintaining this same freedom for others, provided that the original source and author(s) are credited.Daseul Ham, Yeon Jae Bae
